# Electroacupuncture Pretreatment Prevents Cognitive Impairment Induced by Cerebral Ischemia–Reperfusion via Adenosine A1 Receptors in Rats

**DOI:** 10.3389/fnagi.2021.680706

**Published:** 2021-08-03

**Authors:** Yiyi Shi, Qinxue Dai, Binbin Ji, Luping Huang, Xiuxiu Zhuang, Yunchang Mo, Junlu Wang

**Affiliations:** Department of Anesthesiology, The First Affiliated Hospital of Wenzhou Medical University, Wenzhou, China

**Keywords:** EA pretreatment, A1 receptors, cognitive impairment, ischemia–reperfusion, cerebral ischemia

## Abstract

A previous study has demonstrated that pretreatment with electroacupuncture (EA) induces rapid tolerance to focal cerebral ischemia. In the present study, we investigated whether adenosine receptor 1 (A1 R) is involved in EA pretreatment-induced cognitive impairment after focal cerebral ischemia in rats. Two hours after EA pretreatment, focal cerebral ischemia was induced by middle cerebral artery occlusion for 120 min in male Sprague-Dawley rats. The neurobehavioral score, cognitive function [as determined by the Morris water maze (MWM) test], neuronal number, and the Bax/Bcl-2 ratio was evaluated at 24 h after reperfusion in the presence or absence of CCPA (a selective A1 receptor agonist), DPCPX (a selective A1 receptor antagonist) into left lateral ventricle, or A1 short interfering RNA into the hippocampus area. The expression of the A1 receptor in the hippocampus was also investigated. The result showed that EA pretreatment upregulated the neuronal expression of the A1 receptor in the rat hippocampus at 90 min. And EA pretreatment reversed cognitive impairment, improved neurological outcome, and inhibited apoptosis at 24 h after reperfusion. Pretreatment with CCPA could imitate the beneficial effects of EA pretreatment. But the EA pretreatment effects were abolished by DPCPX. Furthermore, A1 receptor protein was reduced by A1 short interfering RNA which attenuated EA pretreatment-induced cognitive impairment.

## Introduction

Stroke is one of the leading causes of morbidity and mortality worldwide, and is a serious threat to human health (Tsai et al., 2013). And cerebral ischemia is one of the main causes accounting for more than 80% of stroke (Goldstein et al., 2001). These patients with cerebral ischemia may have different degrees of memory problems and learning impairment (Longstreth and Dikmen, 1993), and more than 75% of stroke patients suffer from selective cognitive impairment including memory, orientation, language, and attention (Cumming et al., 2013). The hippocampus is the main area for memory and learning behavior in the brain (Lagali et al., 2010), especially, the CA1 region in the hippocampus is so sensitive to ischemia that neurons are easily damaged (Caraci et al., 2008). Although many studies have explored the mechanism of ischemic stroke, the study of cognitive function recovery caused by stroke still needs to be further explored to determine effective treatment.

Adenosine is an essential neuromodulator in the brain, and has been shown to play a role in neuroprotection (Bortolotto et al., 2015). And adenosine functions as a signaling molecule through the activation of four distinct adenosine receptors—denoted as A1, A2A, A2B, and A3 (Cunha, 2001). Adenosine receptors are part of the serine/threonine kinase family and widely expressed in the brain (Lopes et al., 2011). High levels of A1 receptors (A1R) are found in the hippocampus, cortex, and cerebellum, while lower levels are found in the striatum (Shen et al., 2008). It has been shown that adenosine A1 receptors tend to play a role in inhibiting presynaptic nerve activity (Gomes et al., 2011). And adenosine may regulate memory and prevent cognitive impairment (Bortolotto et al., 2015). Adenosine A1 receptors mediate dopamine, glutamate, and BNDF signaling via adenosine, regulate synaptic plasticity in the learning and memory area of brain, and act at a molecular and cellular level to regulate cognitive function (Chen, 2014). In addition, studies about humans and animals support the fact that adenosine receptor activity leads to cognitive enhancement, neuroprotection, and reversal of cognitive impairment in animal models of Alzheimer, Parkinson, Huntington, and schizophrenia (Chen et al., 2014). A subsequent study suggests a more central role for A1R in the selective pattern of neuronal loss in the hippocampus, which is associated with global ischemia (Okamura et al., 2004). The results indicate cognitive impairment caused by cerebral ischemia may be mediated through the adenosine neurotransmitter system (Mioranzza et al., 2011). Therefore, the role of adenosine A1 receptors in cognitive impairment caused by cerebral ischemia has not been studied in depth.

Acupuncture is critical to traditional Chinese medicine, while electroacupuncture (EA) combines traditional Chinese acupuncture and modern electrical techniques. In addition, studies have shown that electroacupuncture pretreatment can mediate cognitive impairment of cerebral ischemia reperfusion injury through the CaM-CaMKIV-CREB (Zhang et al., 2016b) and Wnt pathways (He X. et al., 2016; Chen et al., 2020). Meanwhile, electroacupuncture treatment can prevent the impact of cognitive impairment in the brain, heart, and limbs of ischemia reperfusion patients (Chen et al., 2012; Yuan et al., 2014). Furthermore, several recent studies have reported that A1R may confer acute tolerance to cerebral ischemic/reperfusion injury by electroacupuncture, which plays a neuroprotective role via reducing the release of glutamate (Constantino et al., 2015), limiting postsynaptic depolarization and Ca^2+^ influx (Lubitz et al., 1995). However, the link between electroacupuncture pretreatment effect in cognitive function and adenosine A1 receptors is not entirely clear, and more evidence is needed to prove it.

The aim of our study is to investigate the relationship between electroacupuncture pretreatment and adenosine A1 receptors at pharmacological and genetic levels, and to show that electroacupuncture pretreatment prevents cognitive impairment induced by cerebral ischemia–reperfusion via adenosine A1 receptors.

## Materials and Methods

### Animals

Male Sprague Dawley rats (270–310 g) obtained from the Laboratory Animal Center of Sliaike in Shanghai, China were used in the study and were housed at a constant temperature (24 ± 0.5°C) with a humidity of 55 ± 5% on a controlled 12 h light/dark cycle (light on at 7 a.m.) with free access to food and water. The experimental protocol was approved by the Special Committee on Animal Welfare of Wenzhou Medical University, and all animals were treated humanely according to the National Institutes of Health for Care and Use of Laboratory Animals (NIH Publication No. 85-23, 1996, United States). All efforts were made to minimize animal discomfort and the number of animals used.

### Electroacupuncture Treatment

EA pretreatment was performed as described previously (He X. et al., 2016). Before intraperitoneal injection of chloral hydrate (3 ml/kg), the rats fasted for 12 h. The stainless acupuncture needles (diameter of 0.3 mm) were inserted into the Baihui (GV 20) acupuncture point at a depth of 2–3 mm, which is located at the intersection of the sagittal midline and the line linking the rat ears. Stimulation was generated by the EA apparatus (Model No. 200110510586; Nanjing Jisheng Medical Technology Co., Ltd., Nanjing, China), and the stimulation parameters were set as follows: Disperse wave, 2/15 Hz; electric current, 1 mA; 30 min of each treatment.

### Surgery

Focal cerebral ischemia was induced by middle cerebral artery occlusion (MCAO) using the intraluminal filament technique as previously described (He X. et al., 2016). Two hours after EA pretreatment, the SD rats were deeply anesthetized by chloral hydrate (3 ml/kg). Following exposure of the left common carotid artery (CCA), internal carotid artery (IC), external carotid artery (EC), and proximal branches of the EC, the left MCA was occluded by an insertion of a 0.38 ± 0.02 nylon monofilament suture (Beijing Cinontech Co., Ltd., China) with its tip rounded through the CCA, resulting in the occlusion of the left MCA at its origin. Regional cerebral blood flow was monitored using a transcranial laser Doppler flow meter (PeriFlux5000; Perimed AB, Sweden). MCAO was considered sufficient if the regional cerebral blood flow demonstrated a sharp decrease to 20% of the baseline (pre-ischemic) level; if not, the animal was excluded. Reperfusion was accomplished by withdrawing the suture after 120 min of ischemia. In the group of sham-operated rats, all of the surgical procedures were performed, however, the ICA was not occluded. Following surgery, the rats were transferred to their cage until the animals were completely conscious. Just after reperfusion, the rats were kept in the preoperative state until sampling.

During surgeries for AV-shA1R (1^∗^10^11^ PFU/ml) or AV–shCTRL, 2-chloro-N6-cyclopentyladenosine (0.01 mmol/L, CCPA, Sigma- Aldrich, United States), and 8-cyclopentyl-1, 3-dipropylxanthine (0.01 mmol/L, DPCPX, Sigma-Aldrich, United States) injections were conducted under chloral hydrate (3 ml/kg, ip). Using aseptic techniques, the rats were injected stereotaxically into the left hippocampus at anterior-posterior = -4.80 mm, medial-lateral = 3.20 mm, and dorsal-ventral = -3.2 mm from the bregma or lateral ventricles at anterior-posterior = -0.05 mm, medial-lateral = 1.80 mm, and dorsal-ventral = -4.8 mm from the bregma of the left hemisphere with recombinant. A 33 g needle was inserted under the epineurium of the nerve, 5 μl of virus, CCPA, or DPCPX solution was injected at 0.25 μl/min, using a 10 μl syringe (Hamilton, Switzerland) mounted onto the stereotaxic apparatus and connected to pump. After the injection was completed, the needle was left for 10 min before being removed to stabilize the injection. Then the skin was sutured together and the wound was closed. Animals were allowed to fully recover from the surgery. At 1 h 30 min after the EA treatment, the rats were injected with CCPA or DPCPX and underwent surgery for MCAO. At 48 h after the AV injection, rats underwent surgery for EA and MCAO which were performed as described above.

### Neurobehavioral Evaluation

Twenty-four hours after reperfusion, an observer who was blind to the animal groups assessed the rats using a neurobehavioral test as described previously (Feng et al., 2013). And the scores were determined as follows: Score 0, no neurological deficit; score 1 (failure to fully extend the effect side), mild deficits; scores 2 (circling to the effect side) and 3 (falling to the effect side), moderate deficits; and score 4 (loss of walking), severe deficits.

### Morris Water Maze

The Morris water maze (MWM) test was performed as described previously (Wang et al., 2012) with some modifications. This test was used to assess the effects of EA pretreatment on MCAO-induced learning and memory dysfunctions in rats. This consisted of a circular pool of 100 cm in diameter and 60 cm in height, which was divided factitiously into four equal quadrants (SW, NW, NE, and SE). The pool was filled with water (25 ± 1°C) premixed with black non-toxic paint to make it opaque; a platform (10 cm in diameter) was immersed 2 cm under the surface of the water in one of the four identical quadrants. The pool was located in an illuminated room with some external cues, which remained in the same location throughout the training and testing period.

After induction of global cerebral ischemia for 120 min with EA treatment, each animal was subjected to an acquisition trial and a probe trial. In each acquisition trial, the rats were individually placed in the pool facing the wall at one point randomly selected from different starting points. Rats (*n* = 6) were trained for six blocks on the MWM (three trials per block) 24 h after reperfusion, with a 30-min rest period between trials. During the acquisition trial, the rats were allowed to escape by swimming to the platform and the escape latency was recorded with a cutoff time of 60 s. If the rats failed to locate the platform within 60 s, they were gently guided to the platform and allowed to stay on it for 15 s. Mean escape latency time (MES) to locate the hidden platform in the water maze was recorded as an index of acquisition or learning. Animals that could not swim due to injury following ischemia were eliminated. After the training, the platform was removed from the pool to start the 60 s spatial probe trial test initiated 1 h following the completion of the last trial. Swimming behaviors, including escape latency, swimming speed, and the average time spent in the target quadrant, were monitored using a computer-controlled video-tracking system (CG-400 Image Acquisition System; Institute of Materia Medica, Chinese Academy of Medical Sciences, Shanghai, China). The distance swum, entries and time spent in the target quadrant, and the mean swimming speed were recorded. The mean time taken by the animal searching for the hidden platform in the target quadrant was noted as an index of spatial memory.

### Western Blotting and RT-PCR

To investigate alterations in A1 receptor, Bcl-2, and Bax protein expression, the rats were anesthetized with 10% chloral hydrate (3 ml/kg, ip) and decapitated, the hippocampus of the left hemispheres were dissected and stored at –80°C until analysis. In brief, the brain tissues were sonicated by radioimmunoprecipitation assay lysis buffer (Solarbio, Beijing, China) with phenylmethylsulfonyl fluoride on ice. Tissue extracts were centrifuged at 12,000 × g at 4°C for 15 min. Samples (40 μg of protein each) were separated by electrophoresis in 12% polyacrylamide gels and transferred to a polyvinylidene fluoride (PVDF) membrane. Non-specific bindings were blocked with 5% non-fat dry milk in Tris buffer saline (TBS) in 0.1% Tween-20 at room temperature for 120 min. After washing, membranes were subsequently incubated with respective primary antibodies: Anti-A1 receptor antibody (1:1,000, Abcam, Cambridge, United Kingdom), β-actin polyclonal antibody (1:5,000, Biogot Technology, Co., Ltd., United States), Anti-Bcl-2 antibody (1:1,000, Abcam, Cambridge, United Kingdom) and Anti-Bax antibody (1:5,000, Abcam, Cambridge, United Kingdom) in primary antibody dilution buffer (beyotime, China) at 4°C overnight. Subsequently, the samples were incubated for 1 h at room temperature with horseradish peroxidase-conjugated goat anti-rabbit secondary antibodies (1:5,000, Biogot Technology, Co., Ltd., United States). Membranes were developed by an ECL (Electro-Chemi-Luminescence) technique. The signal intensity of the blots was measured through Image Lab analysis software (Bio-Rad, United States).

Total RNA was extracted from the hippocampus of the left hemispheres with TRIzol reagent (Invitrogen, Carlsbad, CA). The reaction mixture was incubated at 50°C for 30 min for reverse transcription. The continuous amplification program (CFX96 Real-Time PCR Detection System) consisted of one cycle at 95°C for 15 min and 40 cycles at 94°C for 20 s, 60°C for 20 s, and 72°C for 35 s. The expression of GAPDH was used as the internal reference gene and relative quantification was performed using the 2-ΔΔCt method.

### Nissl Staining

Nissl staining was performed to detect neuronal injury. The brain tissue was collected from the rat after removing its head and then embedded with paraffin. Sections were cut to 3 μM thickness using a sliding microtome. Following washing with phosphate-buffered saline (PBS; pH 7.4), tissue sections were dried at 65°C for 2 h, Nissl stained (Leagene, Beijing, China), and dehydrated with 70, 80, 90, and 100% ethanol, respectively, for 5 min each. The sections were cleared in xylene for 5 min twice and finally mounted with neutral balsam (Solarbio, Beijing, China). Stained tissue sections were observed in the hippocampus by light microscopy (BX60; Olympus, Tokyo, Japan).

### Statistical Analysis

Statistical analysis was performed using SPSS 12.0 for Windows (SPSS Inc., Chicago, IL). Data are expressed as the means ± SEM, with the exception of neurobehavioral scores which are expressed as median. All the data were analyzed using one-way analysis of variance (ANOVA) with Fisher’s protected least significance difference test, with the exception of the data of the neurobehavioral scores which were analyzed by the Kruskal-Wallis H test and the data of the water-maze acquisition trials, which were analyzed by multivariate ANOVA with block as a dependent variable, group as a fixed factor, and swimming speed as a covariate followed by Fisher’s least significant difference test. Statistical significance was considered when *P* < 0.05.

## Results

### EA Pretreatment Upregulated A1 Receptor Expression in the Hippocampus

**FIGURE 1:**
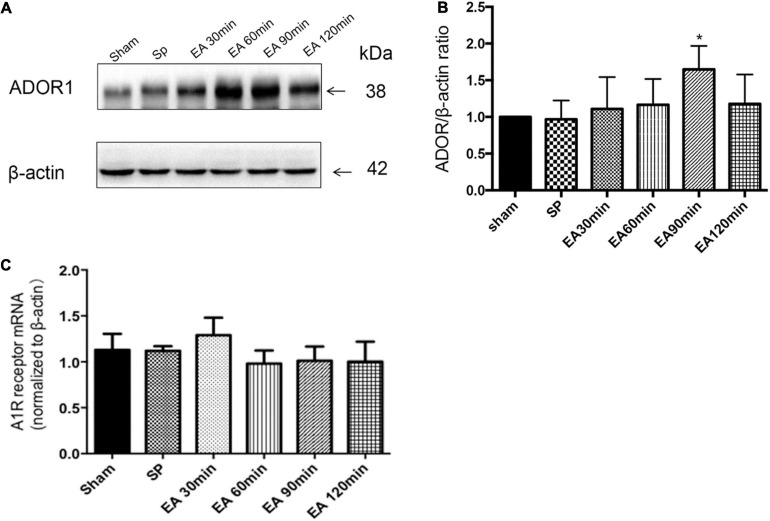
Effects of electroacupuncture (EA) on A1R expression (*n* = 4), as assessed using Western blot and RT-PCR. **(A,B)** Pretreatment with EA significantly increases the expression of A1R protein at 90 min after the completion of EA; **(C)** there is no significant on the expression of A1R mRNA at the times after the completion of EA. *P < 0.01 vs. the sham group.

The effects of EA treatment on A1 receptor expression are shown in Figure 1. The A1 receptor protein expression level 30 min after EA pretreatment increased significantly in the 90 min group compared to the level in the sham group (^∗^*P* < 0.05). There was no significant difference in the level of adenosine A1 receptor mRNA in each time period (*P* > 0.05).

### CCPA Imitated the Effect of EA Pretreatment on Cognitive Function

**FIGURE 2:**
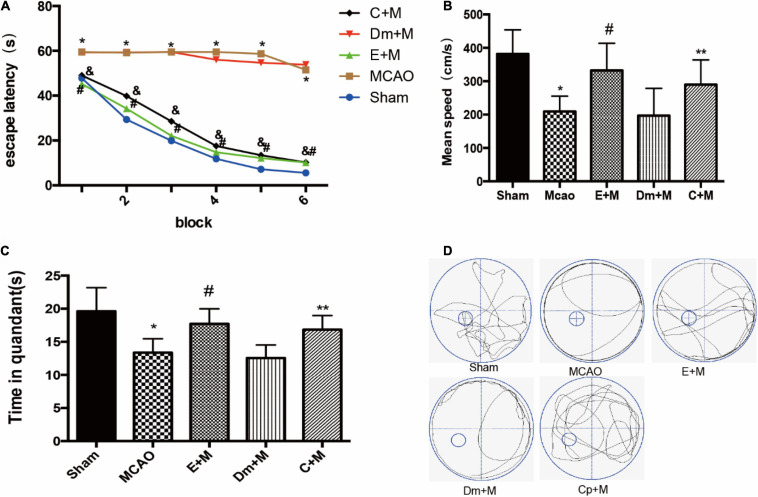
Effects of electroacupuncture (EA) on cognitive function, as assessed using the Morris water maze (MWM) test at 24 h after reperfusion(*n* = 6). **(A)** Changes in the escape latency (i.e., the time required to locate and climb onto the platform) during six-block acquisition trials. EA pretreatment and administration of CCPA decreases the escape latencies in all trials; **(B,C)** effects of EA pretreatment and administration of CCPA on **(B)** mean speed and **(C)** time in quadrant. **(D)** The swimming tracks of rats in different conditions in the probe trial test. *P < 0.01 vs. the sham group, ^#^*P* < 0.05 vs. the MCAO group, **P < 0.05 vs. the MCAO group, ^&^*P* < 0.05 vs. the MCAO group.

To determine whether CCPA and EA treatment reversed cognitive impairment, we examined memory performance in the Morris water-maze test in rats treated with CCPA and EA. Multivariate ANOVA comparisons revealed significant main effects for group (*P* < 0.05), but not for group × swimming speed interaction (*P* > 0.05). Fisher’s least significant difference test revealed reduced escape latencies in the EA + MCAO group compared to the MCAO group in all acquisition trials (EA + MCAO vs. MCAO all *P* < 0.05). Escape latency also reduced in the CCPA + MCAO group (CCPA + MCAO vs. MCAO; all *P* < 0.05). During the probe trials, the CCPA + MCAO group and EA + MCAO group had a significantly longer time spent in the target quadrant and faster swimming speed than the DMSO + MCAO group and MCAO group (Figure 2).

### EA Treatment and CCPA Reduce the Damage in the Hippocampus Following Ischemia–Reperfusion

**FIGURE 3:**
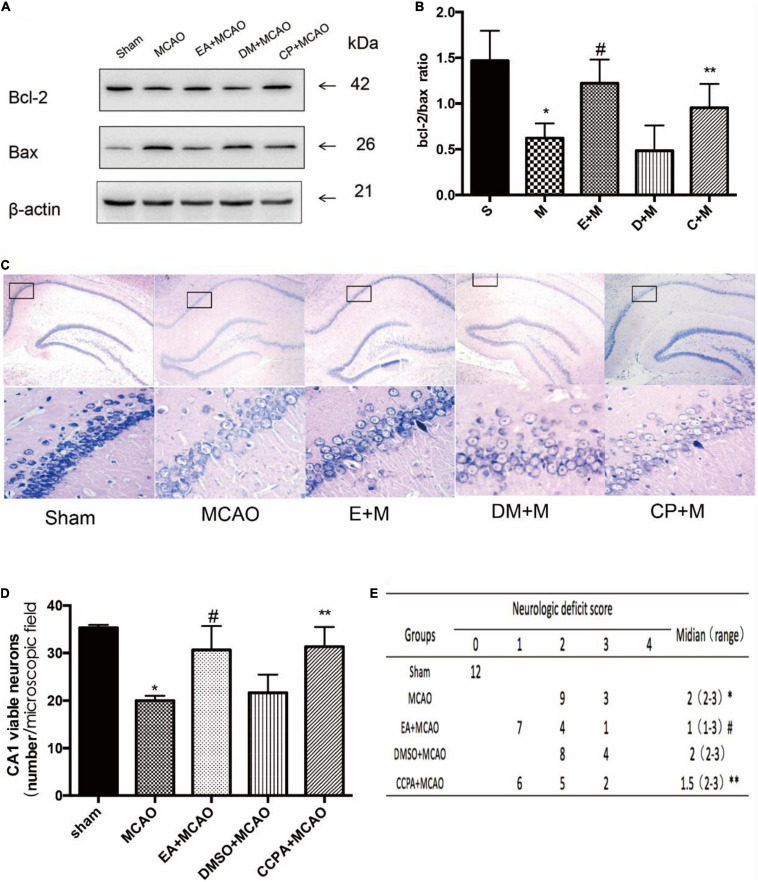
Effect of electroacupuncture (EA) pretreatment and administration of CCPA on the effect of hippocampus damage. **(A,B)** Western blot analysis for Bcl-2 and Bax protein expression in the hippocampus (*n* = 3). **(C,D)** Nissl staining of hippocampus 24 h after reperfusion. Cell counting shows a significant decrease in the number of viable neurons in the hippocampal CA1 region for the middle cerebral artery occlusion (MCAO) group, while the number is significantly increased in the EA + MCAO group and CCPA + MCAO group (*n* = 3). **(E)** Neurologic behavior scores were determined 24 h after reperfusion. *P < 0.05 vs. the sham group, ^#^*P* < 0.05 vs. the MCAO group, **P < 0.05 vs. the MCAO group.

In order to examine the effects of EA treatment and CCPA on nerve function recovery (as shown in Figure 3), we initially investigated the neurological score and the ratio of Bcl-2/Bax which decreased in the MCAO group (Sham vs. MCAO; all *P* < 0.05). But the neurological score and the Bcl-2/Bax ratio was significantly increased in the CCPA and EA-treated rats (EA + MCAO vs. MCAO; CCPA + MCAO vs. MCAO; all *P* < 0.05), suggesting that there is a neuroprotective effect of CCPA and EA against apoptosis in the rat hippocampus. Whereas DMSO pretreatment had no effect on the EA pretreatment (DMSO + MCAO vs. MCAO; *P* > 0.05). These results suggested that EA treatment and CCPA effectively reduce the neurological dysfunction of cerebral ischemia in the hippocampus, promoting nerve functional recovery.

EA pretreatment and CCPA significantly attenuated neuronal loss in the CA1 region of the hippocampus, a feature that was not observed in the MCAO group (EA + MCAO vs. MCAO, *P* < 0.05). No significant differences in the number of viable neurons were detected between the MCAO and DMSO + MCAO groups (DMSO + MCAO vs. MCAO; *P* > 0.05) (Figure 3).

### DPCPX Reversed the Effect of EA Pretreatment on Cognitive Function

**FIGURE 4:**
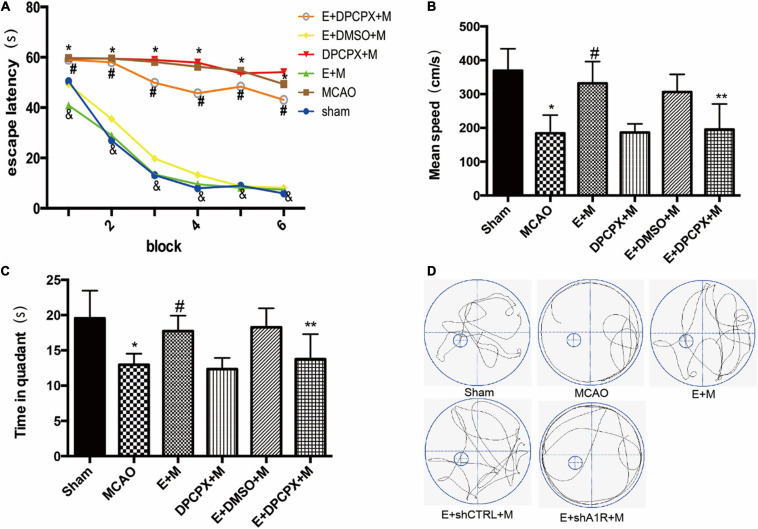
Effects of DPCPX, an adenosine receptor 1 (A1R) antagonist, on cognitive function induced by electroacupuncture (EA) pretreatment (*n* = 6). **(A)** EA pretreatment decreases the escape latencies in all trials; this is reversed by DPCPX; **(B,C)** effects of EA pretreatment with or without DPCPX on **(B)** mean speed and **(C)** time in quadrant. **(D)** The swimming tracks of rats in different conditions in the probe trial test. *P < 0.01 vs. the sham group, ^#^*P* < 0.05 vs. the MCAO group, ^&^*P* < 0.05 vs. the EA + MCAO group, **P < 0.05 vs. the EA + MCAO group.

Whether the cognitive protection of EA pretreatment was reversed by DPCPX pretreatment was examined by the Morris water maze test. The escape latencies in the EA + MCAO group were lower compared to the MCAO group in all acquisition trials (all *P* < 0.05). These findings were reversed by DPCPX (EA + DPCPX + MCAO vs. EA + MCAO, *P* < 0.05; Figure 4). The probe test revealed a significant difference between the EA + DPCPX + MCAO and EA + MCAO groups with regard to swimming speed (*P* < 0.05; Figure 4). We also found that rats with focal cerebral ischemia spent less time in the target quadrant compared to the sham rats (Sham vs. MCAO; *P* < 0.05). EA pretreatment significantly extended the time spent swimming in the target quadrant following MCAO (EA + MCAO vs. MCAO, *P* < 0.05; Figure 4). No significant differences were detected between the MCAO and DPCPX + MCAO groups or between the EA + DMSO + MCAO and EA + MCAO groups (DPCPX + MCAO vs. MCAO; EA + DMSO + MCAO vs. EA + MCAO; all *P* > 0.05).

### DPCPX Reversed the Effect of EA Pretreatment Reducing the Damage in the Hippocampus Following Ischemia–Reperfusion

In order to examine the effects of DPCPX on nerve function recovery, the neurological score and the Bcl-2/Bax ratio were investigated. The MCAO group demonstrated a significant decreased ratio compared with the ratio of the EA + MCAO group (EA + MCAO vs. MCAO, *P* < 0.05). Furthermore, DPCPX reversed the beneficial effects of EA pretreatment (EA + DPCPX + MCAO vs. EA + MCAO, *P* < 0.05), but did not exert an effect when administered alone (MCAO vs. DPCPX + MCAO, *P* > 0.05).

**FIGURE 5:**
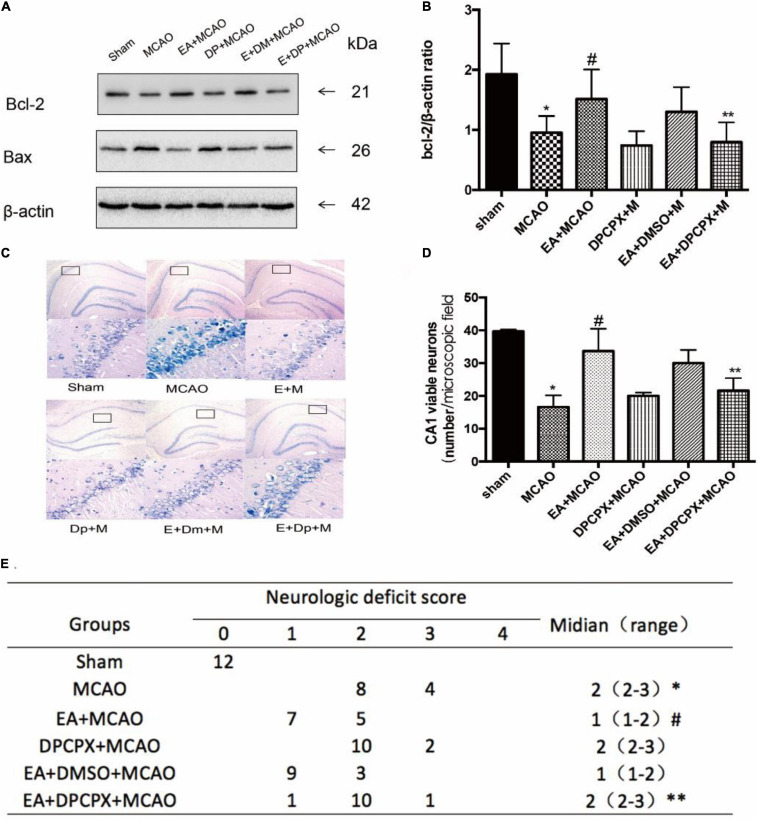
Effect of DPCPX on hippocampus damage induced by electroacupuncture (EA) pretreatment. **(A,B)** Western blot analysis for Bcl-2 and Bax protein expression in the hippocampus (*n* = 3). **(C,D)** EA pretreatment significantly increases the number of viable pyramidal neurons in the hippocampal CA1 region (*n* = 3), whereas DPCPX attenuates these beneficial effects. **(E)** Neurologic behavior scores were determined 24 h after reperfusion. *P < 0.05 vs. the sham group, ^#^*P* < 0.05 vs. the MCAO group, **P < 0.05 vs. the EA + MCAO group.

DPCPX inhibited the beneficial effects of EA pretreatment-attenuated neuronal loss (EA + DPCPX + MCAO vs. EA + MCAO, *P* < 0.05). No significant differences in the number of viable neurons were detected between the MCAO and DPCPX + MCAO groups (MCAO vs. DPCPX + MCAO, *P* > 0.05) (Figure 5).

### The Effect of AV-shA1R in the Hippocampus

**FIGURE 6:**
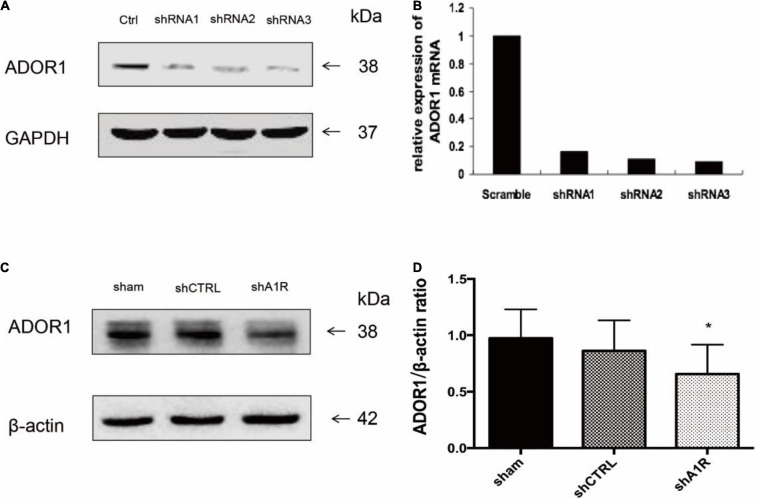
Effect of AV-shRNA on the nerve cells of rats 48 h after administration of AV-shA1R. **(A,B)** Western blot analysis for adenosine receptor 1 (A1R) protein expression and PCR shows A1R mRNA expression (*n* = 3). **(C,D)** A total of 48 h after administration of AV-shA1R and AV–shCTRL on the rats, Western blot analysis for A1R protein expression. *P < 0.05 vs. the sham group.

We examined the effects of AV-shA1R in the hippocampus and found that AV-shRNA3 on the nerve cell was effective. And 48 h after administration of AV-shA1R, the expression of the A1R protein was downregulated in the hippocampus of the rats (shA1R vs. sham, *P* < 0.05) (Figure 6).

### The Effect of AV-shA1R on Cognitive Function

**FIGURE 7:**
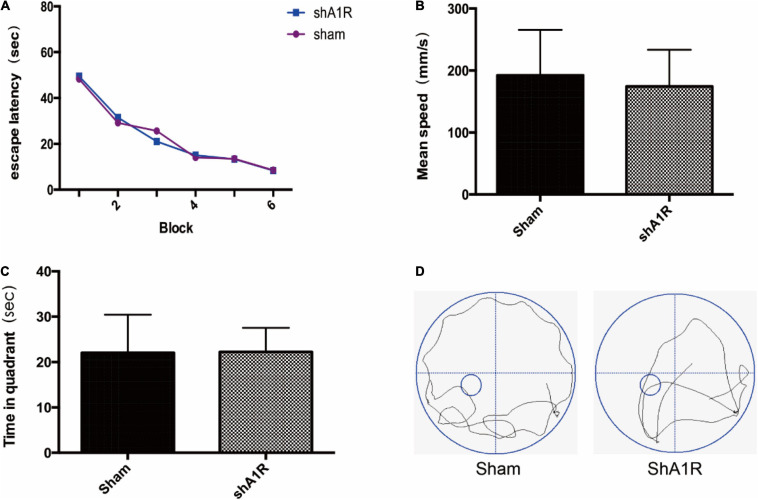
Effects of AV-shA1R on cognitive function (*n* = 6). **(A)** Changes in the escape latency during six-block acquisition trials; **(B,C)** effects of AV-shA1R on **(B)** mean speed and **(C)** time in quadrant. **(D)** The swimming tracks of rats in different conditions in the probe trial test.

In order to examine the effects of AV-shA1R on cognitive function, we examined memory performance in the Morris water maze test in rats treated with AV-shA1R. And we found there was no significant difference between the two groups (*P* > 0.05) (Figure 7).

### AV-shA1R Reversed the Effect of EA Pretreatment on Cognitive Function

**FIGURE 8:**
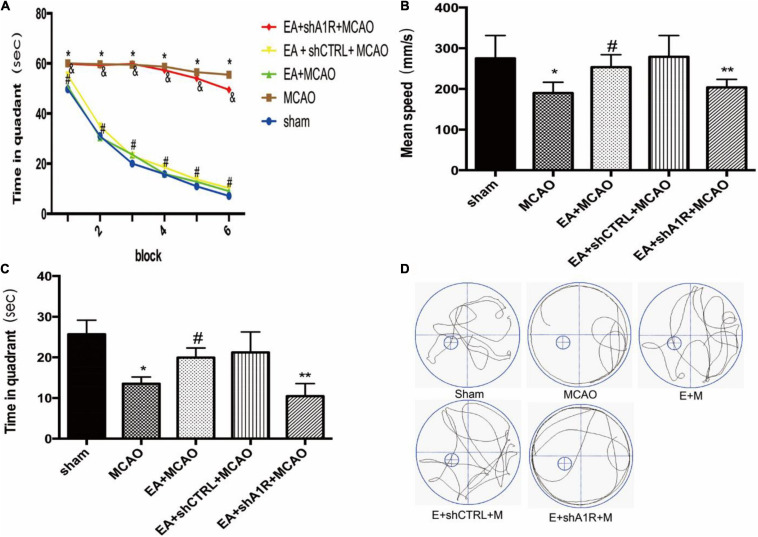
Effects of electroacupuncture (EA) on cognitive function (*n* = 6). **(A)** EA pretreatment decreases the escape latencies in all trials; this is reversed by AV-shA1R; **(B,C)** effects of EA pretreatment with AV-shA1R on **(B)** mean speed and **(C)** time in quadrant. **(D)** The swimming tracks of rats in different conditions in the probe trial test. *P < 0.01 vs. the sham group, ^#^*P* < 0.05 vs. the MCAO group, **P < 0.05 vs. the MCAO group, ^&^*P* < 0.05 vs. the EA + MCAO group, **P < 0.05 vs. the EA + MCAO group.

To determine whether EA pretreatment without the A1 receptor affected cognitive function, we examined memory performance in the Morris water maze test in rats treated with AV-shA1R. As shown in Figure 8, escape latency in the EA + MCAO group was reduced compared with the shA1R + EA + MCAO group (EA + MCAO vs. shA1R + EA + MCAO; all *P* < 0.05) or the MCAO group in all acquisition trials (EA + MCAO vs. MCAO; all *P* < 0.05). During the probe trials, the shA1R + EA + MCAO group had a significantly longer time spent in the target quadrant (EA + MCAO vs. shA1R + EA + MCAO; all *P* < 0.05) (Figure 8). Consistent with this finding, behavioral tracking showed increased exploration time in the target quadrant for the EA + MCAO and EA + shCTRL + MCAO groups compared with the MCAO group. No significant differences were detected between the EA + shCTRL + MCAO and EA + MCAO groups.

### Selective Deletion of A1 Receptor in the Left Hippocampus of Rats Eliminates the Damage in the Hippocampus After EA Pretreatment

**FIGURE 9:**
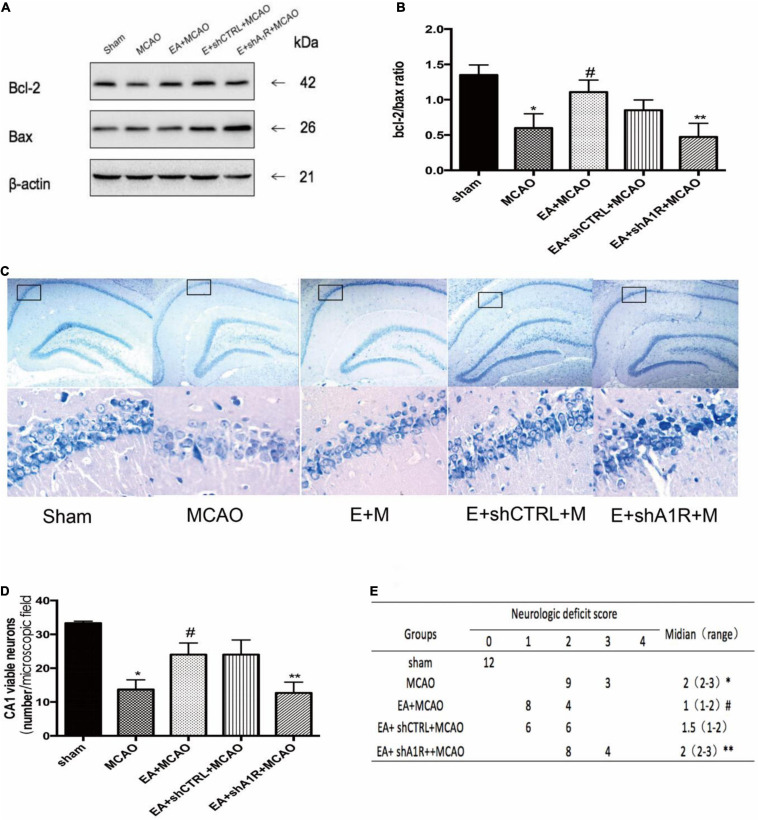
Effect of AV-shA1R on neuroprotection. **(A,B)** Western blot analysis for Bcl-2 and Bax protein expression in the hippocampal CA1 region (*n* = 3). **(C,D)** EA pretreatment significantly increased the number of viable pyramidal neurons in the hippocampal CA1 region (*n* = 3), whereas AV-shA1R attenuated these beneficial effects. **(E)** Neurologic behavior scores were determined 24 h after reperfusion. *P < 0.01 vs. the sham group, ^#^*P* < 0.05 vs. the MCAO group, **P < 0.05 vs. the EA + MCAO group.

Forty-eight hours after administration of AV-shA1R or AV-shCTRL, the western blot result showed that the expression of A1 receptor protein in rats was significantly downregulated in the AV-shA1R administration group (shA1R vs. Sham, shA1R vs. shCTRL; *P* < 0.05), suggesting that the injection sites were on target and AV-shA1R was effective in the rats. The A1R protein deleted by AV-shA1R attenuated the neurological score and the Bcl-2/Bax ratio of EA pretreatment (shA1R + EA + MCAO vs. EA + MCAO, all *P* < 0.05 Figure 9), whereas AV-shCTRL had no effect on EA pretreatment (shCTRL + EA + MCAO vs. EA + MCAO, *P* < 0.05) (Figure 9).

## Discussion

In our study, we found that the A1 receptor protein in the hippocampus was significantly increased at 90 min after electroacupuncture pretreatment of Baihui (GV20). And there was no statistical difference in the levels of adenosine A1 receptor mRNA transcription in each time period. This finding suggests that the adenosine A1 receptor may be involved in the effect of electroacupuncture-preconditioned MCAO model rats. After the subsequent experiments, we explored the hypothesis at the drug level and genetic level. First, we injected the A1 receptor agonist CCPA or A1 receptor antagonist DPCPX into the lateral ventricle of the rats 1 h 30 min after electroacupuncture pretreatment. It was found that A1 receptor activation in MCAO model rats could have the effect of cognitive function recovery. And CCPA imitated the beneficial effects of EA pretreatment. However, DPCPX reversed the protective effect of electroacupuncture. Then we used the transfection technique at the gene level to delete the expression of A1 receptor protein in the hippocampus, which could reverse the protective effect of electroacupuncture. And these results suggest that the A1 receptor is involved in electroacupuncture pretreatment to mediate cognitive impairment after cerebral ischemia, which is a new participatory mechanism.

Previous studies have shown that electroacupuncture pretreatment can be one adjuvant therapy of stroke, and the prospect is considerable (Gosman-Hedstrom et al., 1998). Pretreatment with EA at the Baihui acupoint (GV 20) induces a rapid tolerance 2 h after EA to cerebral ischemic insults (Zhou et al., 2013). And electroacupuncture mediates through the CB1 receptor (Wang et al., 2009; Du et al., 2010; Ma et al., 2011), Wnt signaling pathway (He X. et al., 2016), and an adenosine A1 receptor-related mechanism against transient cerebral ischemia, by inhibiting apoptosis, producing antioxidant protection, reducing inflammatory mediators, and reducing excitotoxicity to produce brain protection (Shen et al., 2016). In addition, these studies have shown that hippocampus ischemic tolerance is associated with GluR2 elevating after electroacupuncture pretreatment (Liu et al., 2015). And some studies have shown that electroacupuncture pretreatment for hippocampus-related diseases resulted in convalescence (He X. L. et al., 2016; Wang et al., 2016; Liu et al., 2017).

The hippocampus, an important part in the limbic system, is not only very sensitive to cerebral ischemia–reperfusion injury (Lee et al., 1986), where the vertebral neurons of the CA1 region are sensitive and fragile to harmful injury (Caraci et al., 2008), but also plays a major role in the formation and consolidation of learning and memory (Lagali et al., 2010; Bartsch and Wulff, 2015). Simultaneously, the destruction in the hippocampal CA1 area is associated with cognitive impairment (Zhang et al., 2016b). So, the hippocampus is the main tissue to explore in our research. The study has demonstrated that after electroacupuncture pretreatment, the neurobehavioral score was improved, the escape latency was decreased, and target quadrant was increased in the MCAO rats, as in a previous study (He X. et al., 2016). And we also found that after CCPA administration in the MCAO rats, the behavioral score and the results in the water maze test were comparable to electroacupuncture pretreatment, but the effect was reversed after DPCPX administration.

CCPA is an agonist of the adenosine A1 receptor (Cristalli et al., 1986) and is used in A1 receptor-related experiments (Lohse et al., 1988). CCPA administration has protective effects on ischemic neuronal injury (Goda et al., 1998). DPCPX is an adenosine A1 receptor antagonist (Okamura et al., 2004) and is also used in A1 receptor-related experiments. DPCPX administration reduces neuroprotective effects (Yoshida et al., 2004) and attenuates cerebral ischemic tolerance (Nakamura et al., 2002). The adenosine-related drugs are used for the treatment of human cognitive and memory-related pathologies, which is used to treat schizophrenia, panic disorder, and anxiety (Lopes et al., 2011). And other studies show that after cerebral ischemia, A1 receptors attempt to inhibit presynaptic glutamate release and to limit postsynaptic depolarization and Ca^2+^ influx (Lubitz, 1998). In this study, we found that the A1 receptor protein in the hippocampus was significantly increased 90 min after electroacupuncture pretreatment. And under hypoxic conditions, the A1 receptor density in the hippocampus was decreased (Lee et al., 1986). The result suggests that A1 receptors may be involved in the electroacupuncture pretreatment mechanism. Meanwhile A1 receptors are associated with neuroprotection (Fedele et al., 2006), and the A1 receptor is necessary to mediate cerebral ischemic tolerance in ischemic conditions (Yoshida et al., 2004). Electroacupuncture has now been used for cerebral ischemic disease (Gao et al., 2006; Wang et al., 2009), and studies have shown that electroacupuncture may stimulate adenosine A1 receptors to mediate rapid cerebral ischemic tolerance and cognitive function (Zhang et al., 2016b). In order to explore the association between A1 receptors and electroacupuncture, CCPA and DPCPX were injected into the lateral ventricle in the rats. We confirmed that CCPA can replicate the effect of electroacupuncture to inhibit neuronal apoptosis in the MCAO rats, whereas DPCPX can reverse this effect, as in a previous study. We also found that electroacupuncture pretreatment stimulates A1 receptors to inhibit neuronal apoptosis and play a protective role in cognitive function after cerebral ischemia, whereas DPCPX can also reverse this protective effect and aggravate cerebral ischemic injury. So, these results suggest that the A1 receptor is involved in electroacupuncture preconditioning-induced cognitive recovery after cerebral ischemia. To further confirm that, we used the transfection technique to silence the A1 receptor. The adenovirus with the shA1R sequence downregulated the expression of A1 receptor protein in the hippocampus. And the cognitive protection effect of electroacupuncture pretreatment was inhibited after the A1 receptor was downregulated. These findings further elucidate that the protective effect on cognitive function induced by electroacupuncture pretreatment is related to the A1 receptor-related signaling pathway.

Cerebral injury is caused by ischemia and hypoxia mainly in the CA1 area of the hippocampus; the hippocampus was the focus in our research. And we found that the memory of animals with hippocampal damage was impaired in the Morris water maze test (Boissardm et al., 1992). So, we chose the Morris water maze to detect the learning and memory function of SD rats. In our study, the memory of AV-shA1R rats and sham-operated rats was no different, indicating that the learning and memory function of rats with downregulated A1 receptors is unchanged. A previous study (Lang et al., 2003) confirmed that the spatial learning ability and exploratory ability of A1 receptor-knockout mice were at normal levels. In our study, the transfection technique reduced the expression of A1 receptor protein in the hippocampus, which reversed the effect of pretreatment electroacupuncture in the cognitive impairment induced by cerebral ischemia–reperfusion in the rats. For further study, the expression of A1 receptor protein can be overexpressed by the transfection technique in the hippocampus, and the cognitive impairment induced by cerebral ischemia–reperfusion in rats can be observed. And cognitive impairment caused by hippocampal injury can also be tested by a light and dark shuttle test (Wang et al., 2012; Wang et al., 2020) and other tests. The study needs more cognitive behavioral tests to explore cognitive function. Because Bcl protein and Bax protein activity are critical to the activation and inhibition of apoptosis and may be associated with the mechanism of brain injury (Goda et al., 1998), we assessed the ischemic injury of the hippocampus by Bcl-2/Bax ratio and the localization of the nucleus and the type and the distribution of neurons by Nissl staining (Zhang et al., 2016a). We used Nissl staining to determine the viable neurons in the CA1 region of the hippocampus. In the end, the results suggest that electroacupuncture pretreatment had an effect on the learning and memory dysfunction of rats, which reduced the degree of hippocampal injury.

Our study demonstrates that electroacupuncture pretreatment can prevent cognitive impairment from cerebral ischemia–reperfusion, in which the effect is mediated through the A1 receptor. The mechanism of electroacupuncture pretreatment reduces cognitive dysfunction by reducing neuronal damage in the hippocampus. The A1 receptor has neuroprotective effects due to adenosine in presynaptic receptors, reducing excitatory neurotransmitters and the activation of postsynaptic receptors that results in K^+^ channel hyperpolarization (Ciruela et al., 2012). Previous studies have shown that adenosine of adult animals can inhibit NMDA receptor activation and reduce Ca^2+^ influx to reduce excitotoxicity (Cunha, 2005). And A1 receptor activation has an effect on NMDA receptors to reduce harmful substance release (Constantino et al., 2015). Meanwhile A1 receptors may have an effect on Glu A2 to reduce hippocampal injury after ischemia (Liu et al., 2015; Stockwell et al., 2016). And whether these mechanisms are also involved in A1 receptor-mediated cognitive impairment with cerebral ischemia by electroacupuncture preconditioning requires exploration.

## Data Availability Statement

The original contributions presented in the study are included in the article/supplementary material, further inquiries can be directed to the corresponding author/s.

## Ethics Statement

The animal study was reviewed and approved by the Special Committee on Animal Welfare of Wenzhou Medical University. Written informed consent was obtained from the owners for the participation of their animals in this study.

## Author Contributions

JW and YS designed the study. YS and QD implemented the experimental design and participated in data collection and analysis. BJ, LH, and YM developed the clinical protocols. YS and XZ drafted the manuscript. All authors read and approved the final manuscript.

## Conflict of Interest

The authors declare that the research was conducted in the absence of any commercial or financial relationships that could be construed as a potential conflict of interest.

## Publisher’s Note

All claims expressed in this article are solely those of the authors and do not necessarily represent those of their affiliated organizations, or those of the publisher, the editors and the reviewers. Any product that may be evaluated in this article, or claim that may be made by its manufacturer, is not guaranteed or endorsed by the publisher.

## References

[B1] Bartsch T. Wulff P. (2015). The hippocampus in aging and disease: from plasticity to vulnerability. *Neuroscience* 309 1–16. 10.1016/j.neuroscience.2015.07.084 26241337

[B2] Boissardm C. G. Lindner M. D. Gribkoff V. K. (1992). Hypoxia produces cell death in the rat hippocampus in thepresence of an A1 adenosine receptor antagonist an anatomical and behavioral study. *Neuroscience* 48 807–812. 10.1016/0306-4522(92)90268-7 1630626

[B3] Bortolotto J. W. Melo G. M. Cognato G. D. P. Vianna M. R. Bonan C. D. (2015). Modulation of adenosine signaling prevents scopolamine-induced cognitive impairment in zebrafish. *Neurobiol. Learn. Mem.* 118 113–119. 10.1016/j.nlm.2014.11.016 25490060

[B4] Caraci F. Busceti C. Biagioni F. Aronica E. Mastroiacovo F. Cappuccio I. (2008). The Wnt antagonist, Dickkopf-1, as a target for the treatment of neurodegenerative disorders. *Neurochem. Res.* 33 2401–2406. 10.1007/s11064-008-9710-0 18427981

[B5] Chen C. Yu Q. Xu K. Cai L. Felicia B. M. Wang L. (2020). Electroacupuncture pretreatment prevents ischemic stroke and inhibits Wnt signaling-mediated autophagy through the regulation of GSK-3β phosphorylation. *Brain Res. Bull.* 158 90–98. 10.1016/j.brainresbull.2020.03.002 32142833

[B6] Chen J. F. (2014). Adenosine receptor control of cognition in normal and disease. *Int. Rev. Neurobiol.* 119 257–307. 10.1016/B978-0-12-801022-8.00012-X 25175970

[B7] Chen J. F. Lee C. F. Chern Y. (2014). Adenosine receptor neurobiology: overview. *Int. Rev. Neurobiol.* 119 1–49. 10.1016/B978-0-12-801022-8.00001-5 25175959

[B8] Chen Y. Zhou J. Li J. Yang S. B. Mo L. Q. Hu J. H. (2012). Electroacupuncture pretreatment prevents cognitive impairment induced by limb ischemia-reperfusion via inhibition of microglial activation and attenuation of oxidative stress in rats. *Brain Res.* 1432 36–45. 10.1016/j.brainres.2011.11.002 22129788

[B9] Ciruela F. Fernandez-Duenas V. Llorente J. Borroto-Escuela D. Cuffi M. L. Carbonell L. (2012). G protein-coupled receptor oligomerization and brain integration: focus on adenosinergic transmission. *Brain Res.* 1476 86–95. 10.1016/j.brainres.2012.04.056 22575562

[B10] Constantino L. C. Pamplona F. A. Matheus F. C. Ludka F. K. Gomez-Soler M. Ciruela F. (2015). Adenosine A1 receptor activation modulates N-methyl-d-aspartate (n.d.) preconditioning phenotype in the brain. *Behav. Brain Res.* 282 103–110. 10.1016/j.bbr.2014.12.056 25557798

[B11] Cristalli G. Grifantini M. Vittori S. Klotz K.-N. Lohse M. J. (1986). Synthesis of 2-azido-(R)-N6-p-hydroxyphenylisopropy-l adenosine (R-AHPIA) as a potential photoaffinity probe for AI adenosine receptors. *Nucleos. Nucleot.* 5 213–222. 10.1080/07328318608068674

[B12] Cumming T. B. Marshall R. S. Lazar R. M. (2013). Stroke, cognitive deficits, and rehabilitation: still an incomplete picture. *Int. J. Stroke* 8 38–45. 10.1111/j.1747-4949.2012.00972.x 23280268

[B13] Cunha R. A. (2001). Adenosine as a neuromodulator and as a homeostatic regulator in the nervous system: different roles, different sources and different receptors. *Neurochem. Int.* 38 107–125. 10.1016/s0197-0186(00)00034-6 11137880

[B14] Cunha R. A. (2005). Neuroprotection by adenosine in the brain: from A(1) receptor activation to A (2A) receptor blockade. *Purinergic Signal.* 1 111–134. 10.1007/s11302-005-0649-1 18404497 PMC2096528

[B15] Du J. Wang Q. Hu B. Peng Z. Zhao Y. Ma L. (2010). Involvement of ERK 1/2 activation in electroacupuncture pretreatment via cannabinoid CB1 receptor in rats. *Brain Res.* 1360 1–7. 10.1016/j.brainres.2010.07.034 20654595

[B16] Fedele D. E. Li T. Lan J. Q. Fredholm B. B. Boison D. (2006). Adenosine A1 receptors are crucial in keeping an epileptic focus localized. *Exp. Neurol.* 200 184–190. 10.1016/j.expneurol.2006.02.133 16750195

[B17] Feng X. Yang S. Liu J. Huang J. Peng J. Lin J. (2013). Electroacupuncture ameliorates cognitive impairment through inhibition of NF-kappaB-mediated neuronal cell apoptosis in cerebral ischemia-reperfusion injured rats. *Mol. Med. Rep.* 7 1516–1522. 10.3892/mmr.2013.1392 23525450

[B18] Gao H. Guo J. Zhao P. Cheng J. (2006). Influences of electroacupuncfure on the expression of insulin-like growth factor-1 following focal cerebral ischemia in monkeys. *Acupunct. Electro Ther.* 31 259–272. 10.3727/036012906815844247 17608065

[B19] Goda H. Ooboshi H. Nakane H. Ibayashi S. Sadoshima S. Fujishima M. (1998). Modulation of ischemia-evoked release of excitatory andinhibitory amino acids by adenosine A1 receptor agonist. *Eur. J. Pharmocol.* 357 149–155. 10.1016/s0014-2999(98)00559-7 9797030

[B20] Goldstein L. B. Adams R. Becker K. Furberg C. D. Gorelick P. B. Hademenos G. (2001). Primary prevention of ischemic stroke: a statement for healthcare professionals from the stroke council of the american heart association. *Circulation* 103 163–182. 10.1161/01.cir.103.1.163 11136703

[B21] Gomes C. V. Kaster M. P. Tome A. R. Agostinho P. M. Cunha R. A. (2011). Adenosine receptors and brain diseases: neuroprotection and neurodegeneration. *Biochim. Biophys. Acta* 1808 1380–1399. 10.1016/j.bbamem.2010.12.001 21145878

[B22] Gosman-Hedstrom G. Claesson L. Klingenstierna U. Carlsson J. Olausson B. Frizell M. (1998). Effects of acupuncture treatment on daily life activities and quality of life: a controlled, prospective, and randomized study of acute stroke patients. *Stroke* 29 2100–2108. 10.1161/01.str.29.10.2100 9756589

[B23] He X. L. Zhao S. H. You W. Cai Y. Y. Wang Y. Y. Ye Y. M. (2016). Neuroprotective effects of electroacupuncture preventive treatment in senescence-accelerated mouse prone 8 mice. *Chin. J. Integr. Med*. 24 133–139. 10.1007/s11655-016-2265-z 27670874

[B24] He X. Mo Y. Geng W. Shi Y. Zhuang X. Han K. (2016). Role of Wnt/beta-catenin in the tolerance to focal cerebral ischemia induced by electroacupuncture pretreatment. *Neurochem. Int.* 97 124–132. 10.1016/j.neuint.2016.03.011 26994873

[B25] Lagali P. S. Corcoran C. P. Picketts D. J. (2010). Hippocampus development and function: role of epigenetic factors and implications for cognitive disease. *Clin. Genet.* 78 321–333. 10.1111/j.1399-0004.2010.01503.x 20681996

[B26] Lang U. E. Lang F. Richter K. Vallon V. Lipp H. P. Schnermann J. (2003). Emotional instability but intact spatial cognition inadenosine receptor 1 knock out mice. *Behav. Brain Res.* 145 179–188. 10.1016/S0166-4328(03)00108-6 14529816

[B27] Lee K. S. Tetzlaff W. Kreutzberg G. W. (1986). Rapid down regulation of hippocampal adenosine receptors following brief anoxia. *Brain Res.* 380 155–158. 10.1016/0006-8993(86)91440-x 3756467

[B28] Liu W. Wu J. Huang J. Zhuo P. Lin Y. Wang L. (2017). Electroacupuncture regulates hippocampal synaptic plasticity via miR-134-mediated LIMK1 function in rats with ischemic stroke. *Neural Plast.* 2017:9545646. 10.1155/2017/9545646 28116173 PMC5237739

[B29] Liu Z. Chen X. Gao Y. Sun S. Yang L. Yang Q. (2015). Involvement of GluR2 up-regulation in neuroprotection by electroacupuncture pretreatment via cannabinoid CB1 receptor in mice. *Sci. Rep.* 5:9490. 10.1038/srep09490 25830356 PMC4381620

[B30] Lohse M. J. Klotz K.-N. Schwabe U. Cristalli G. Vittoriz S. Grifantini M. (1988). 2-chloro-N6-cyclopentyladenosine a highly selectiveagonist at A1 adenosine receptors. *Naunyn Schmiedeberg’s Arch. Pharmacol.* 337 687–689. 3216901 10.1007/BF00175797

[B31] Longstreth W. T. Jr. Dikmen S. S. (1993). Outcomes after cardiac arrest. *Ann. Emerg. Med.* 22 64–69. 8424617 10.1016/s0196-0644(05)80252-5

[B32] Lopes L. V. Sebastião A. M. Ribeiro J. A. (2011). Adenosine and related drugs in brain diseases present andfuture in clinical trials. *Curr. Top. Med. Chem.* 11 1087–1101. 10.2174/156802611795347591 21401493

[B33] Lubitz D. K. J. E. V. (1998). Adenosine and cerebral ischemia therapeutic future ordeath of a brave concept. *Eur. J. Pharmacol.* 371 85–102. 10.1016/s0014-2999(99)00135-1 10355598

[B34] Lubitz D. K. V. Lin R. C. Jacobson K. A. (1995). Jacobson, cerebral ischemia in gerbils: effects of acute and chronic treatment with adenosine A2A receptor agonist and antagonist. *Eur. J. Pharmacol.* 287 295–302. 10.1016/0014-2999(95)00498-x 8991804 PMC4827157

[B35] Ma L. Zhu Z. Zhao Y. Hou L. Wang Q. Xiong L. (2011). Cannabinoid receptor type 2 activation yields delayed tolerance to focal cerebral ischemia. *Curr. Neurovasc. Res.* 8 145–152. 10.2174/156720211795495394 21443454

[B36] Mioranzza S. Costa M. S. Botton P. H. Ardais A. P. Matte V. L. Espinosa J. (2011). Blockade of adenosine A(1) receptors prevents methylphenidate-induced impairment of object recognition task in adult mice. *Prog. Neuropsychopharmacol. Biol. Psychiatry* 35 169–176. 10.1016/j.pnpbp.2010.10.022 21044657

[B37] Nakamura M. Nakakimura K. Matsumoto M. Sakabe T. (2002). Rapid tolerance to focal cerebral ischemia in rats is attenuated by adenosine A1 receptor antagonist. *J. Cereb. Blood Flow Metab.* 22 161–170. 10.1097/00004647-200202000-00004 11823714

[B38] Okamura N. Hashimoto K. Shimizu E. Kumakiri C. Komatsu N. Iyo M. (2004). Adenosine A1 receptor agonists block the neuropathological changes in rat retrosplenial cortex after administration of the NMDA receptor antagonist dizocilpine. *Neuropsychopharmacology* 29 544–550. 10.1038/sj.npp.1300351 14603270

[B39] Shen H. Y. Coelho J. E. Ohtsuka N. Canas P. M. Day Y. J. Huang Q. Y. (2008). A critical role of the adenosine A2A receptor in extrastriatal neurons in modulating psychomotor activity as revealed by opposite phenotypes of striatum and forebrain A2A receptor knock-outs. *J. Neurosci.* 28 2970–2975. 10.1523/JNEUROSCI.5255-07.2008 18354001 PMC6670718

[B40] Shen M. H. Zhang C. B. Zhang J. H. Li P. F. (2016). Electroacupuncture attenuates cerebral ischemia and reperfusion injury in middle cerebral artery occlusion of rat via modulation of apoptosis, inflammation, oxidative stress, and excitotoxicity. *Evid. Based Complement. Alternat. Med.* 2016:9438650. 10.1155/2016/9438650 27123035 PMC4830716

[B41] Stockwell J. Chen Z. Niazi M. Nosib S. Cayabyab F. S. (2016). Protein phosphatase role in adenosine A1 receptor-induced AMPA receptor trafficking and rat hippocampal neuronal damage in hypoxia/reperfusion injury. *Neuropharmacology* 102 254–265. 10.1016/j.neuropharm.2015.11.018 26626486

[B42] Tsai C.-F. Thomas B. Sudlow C. L. M. (2013). Epidemiology of stroke and its subtypes in Chinese vs white population. *Neurology* 81 264–272. 10.1212/wnl.0b013e31829bfde3 23858408 PMC3770160

[B43] Wang C. Yang X. M. Zhuo Y. Y. Zhou H. Lin H. B. Cheng Y. F. (2012). The phosphodiesterase-4 inhibitor rolipram reverses Abeta-induced cognitive impairment and neuroinflammatory and apoptotic responses in rats. *Int. J. Neuropsychopharmacol.* 15 749–766. 10.1017/S1461145711000836 21733236

[B44] Wang J. Li P. Qin T. Sun D. Zhao X. Zhang B. (2020). Protective effect of epigallocatechin-3-gallate against neuroinflammation and anxiety-like behavior in a rat model of myocardial infarction. *Brain Behav.* 10:e01633. 10.1002/brb3.1633 PMC7303397 32304289

[B45] Wang Q. Peng Y. Chen S. Gou X. Hu B. Du J. (2009). Pretreatment with electroacupuncture induces rapid tolerance to focal cerebral ischemia through regulation of endocannabinoid system. *Stroke* 40 2157–2164. 10.1161/STROKEAHA.108.541490 19372445

[B46] Wang X. Huang Y. Yuan S. Tamadon A. Ma S. Feng Y. (2016). The role of hippocampal estradiol receptor-alpha in a perimenopausal affective disorders-like rat model and attenuating of anxiety by electroacupuncture. *Evid. Based Complement. Alternat. Med.* 2016:4958312. 10.1155/2016/4958312 28044085 PMC5156811

[B47] Yoshida M. Nakakimura K. Cui Y. J. Matsumoto M. Sakabe T. (2004). Adenosine A(1) receptor antagonist and mitochondrial ATP-sensitive potassium channel blocker attenuate the tolerance to focal cerebral ischemia in rats. *J. Cereb. Blood Flow Metab.* 24 771–779. 10.1097/01.WCB.0000122742.72175.1B 15241185

[B48] Yuan S. Zhang X. Bo Y. Li W. Zhang H. Jiang Q. (2014). The effects of electroacupuncture treatment on the postoperative cognitive function in aged rats with acute myocardial ischemia-reperfusion. *Brain Res.* 1593 19–29. 10.1016/j.brainres.2014.10.005 25446007

[B49] Zhang Y. Jia X. Yang J. Li Q. Yan G. Xu Z. (2016a). Effects of Shaoyao-Gancao decoction on infarcted cerebral cortical neurons: suppression of the inflammatory response following cerebral ischemia-reperfusion in a rat model. *Biomed. Res. Int.* 2016:1859254. 10.1155/2016/1859254 27413737 PMC4931082

[B50] Zhang Y. Lin R. Tao J. Wu Y. Chen B. Yu K. (2016b). Electroacupuncture improves cognitive ability following cerebral ischemia reperfusion injury via CaM-CaMKIV-CREB signaling in the rat hippocampus. *Exp. Ther. Med.* 12 777–782. 10.3892/etm.2016.3428 27446275 PMC4950700

[B51] Zhou H. Zhang Z. Wei H. Wang F. Guo F. Gao Z. (2013). Activation of STAT3 is involved in neuroprotection by electroacupuncture pretreatment via cannabinoid CB1 receptors in rats. *Brain Res.* 1529 154–164. 10.1016/j.brainres.2013.07.006 23880371

